# Trends in Thinness, Overweight, and Obesity Among Chinese Children Aged 2–18 Years Before and During the COVID-19 Pandemic in 2010–2020

**DOI:** 10.2188/jea.JE20250061

**Published:** 2025-12-05

**Authors:** Chengyue Li, Shuai Zhang, Zhidong Zhou, Jianhua Zhang

**Affiliations:** 1 School of Physical Education and Art, Hunan University of Medicine, Huaihua, China; 2 School of Social Sports, Tianjin University of Sport, Tianjin, China; 3 Institute of Physical Education, Xinjiang Normal University, Urumqi, China; 4 School of Sports Science, Jishou University, Jishou, China

**Keywords:** child, thinness, overweight, obesity, trend, COVID-19

## Abstract

**Background:**

Changes in nutritional status during the coronavirus disease 2019 (COVID-19) pandemic may be attributed to the obesity-causing environment that had existed before. This paper aimed to investigate trends in thinness, overweight, and obesity among Chinese children aged 2 to 18 years from 2010 to 2020 and assess the potential influence of pandemic.

**Methods:**

The Chinese Family Panel Studies that were conducted every 2 years between 2010 and 2020 included 48,642 children between the ages of 2 and 18 years. Height and mass were reported, and the body mass index (BMI) was calculated. The prevalence of thinness, overweight, and obesity was estimated using sex- and age-specific BMI cut-offs adopted by the International Obesity Task Force and population-weighted procedures. Linear regressions were used to estimate trends.

**Results:**

The prevalence of thinness decreased from 25.5% to 22.2% but increased among children aged 2 to 6 years. The prevalence of overweight and obesity decreased from 24.6% and 14.2% to 22.5% and 10.0%, respectively. However, these decreases in the prevalence of overweight and obesity were attributed mostly to children aged 2 to 6 years, and the prevalence increased slightly among children aged 13 to 18 years. The prevalence of obesity in 2020 among only boys aged 13 to 15 years was greater than the estimated projections using data from 2010 to 2018.

**Conclusion:**

From 2010 to 2020, the prevalence of all forms of malnutrition among Chinese children decreased, with age disparities. During the pandemic, overweight became more common among pubertal boys. Future interventions as well as policies ought to give high-risk groups priority.

## INTRODUCTION

All forms of malnutrition are among the greatest global social challenges and serious public health problems worldwide.^1^^,^^2^ Childhood malnutrition phenotypes (ie, thinness, overweight, and obesity) predict adverse health trajectories across the life course, with excessive adiposity acquisition exhibiting high tracking stability into adulthood.^3^^–^^5^ In addition to correcting some malnutrition and imbalances, a healthy developmental environment also helps strengthen the initial developmental outcomes in childhood.^4^^,^^6^ Therefore, for children to make a healthy transition to adulthood, appropriate measures to protect their nutritional status are crucial.

Global prevalence of obesity in children increased from 0.7% in 1975 to 5.6% in 2016 and 8% in 2022, according to the Non-Communicable Disease Risk Factor Collaboration surveys, which pooled surveys from around the world. At the same time, the prevalence of thinness declined gradually, resulting in an increase in malnutrition.^2^^,^^3^ Since the 21st century, body mass index (BMI) and the detection rates of obesity have increased little in high-income countries but have increased rapidly in some emerging economies.^1^^,^^2^^,^^4^ Over the past 30 years, the primary nutritional issues affecting children in China have changed from thinness to obesity or overweight.^7^^,^^8^ Some recent reviews and surveys reported that the rapid prevalence of overweight and obesity has been curbed, with detection rates stabilizing or declining in recent years.^9^^–^^11^ In light of the possibility that China still faces the double malnutrition burden, there is a dearth of data regarding nationally representative tendencies, particularly about thinness.^5^^,^^7^

The global coronavirus disease 2019 (COVID-19) pandemic began in December 2019. To contain the spread of COVID-19, the government has taken strong measures, such as lockdowns, school closures, and home quarantines. Following intensive containment measures, sustained control of the community pandemic was established across China by April 2020, transitioning to routine prevention protocols. Compulsory measures during this period may have altered children’s lifestyles and exacerbated the generally overweight- and obesity-causing environments.^12^ The prevalence of obesity rose during the pandemic, with the most pronounced increase observed after initial lockdown measures in children.^12^^,^^13^ Nevertheless, a reversal trend emerged during prolonged prevention efforts, with declining overweight or obesity burdens.^13^^,^^14^ Unfortunately, these studies were conducted over a short time span and did not adequately consider the impact of these measures on long-term trends in weight status, and changes in body mass during the pandemic may have been related to the obesity-causing environment that had existed before.

Therefore, based on the Chinese Family Panel Studies (CFPS) data in 2010–2020, this paper aimed to investigate (1) trends in the prevalence of thinness, overweight, and obesity; (2) sex and age differences in all malnutrition types; and (3) the effect of the COVID-19 pandemic on weight status among Chinese children aged 2–18 years.

## METHODS

### Study design and population

The CFPS baseline survey (2010) employed a multi-stage probability sampling strategy with implicit stratification and population-proportional allocation. This framework initially covered 16,000 households in 25 autonomous/provinces/municipalities (excluding Qinghai, Tibet, Xinjiang, Inner Mongolia, Ningxia, Hong Kong, Hainan, Taiwan, and Macao). Except for Macao, Taiwan, and Hong Kong, the population of the 25 provincial areas makes up about 95% of the nation’s population, making it a countrywide representative sample. The sampling involved: administrative districts/counties, administrative villages/committees, and households. The detailed process has been defined before.^5^^,^^15^ Since 2010, the CFPS has been tracked every 2 years. Therefore, data from six surveys in 2010, 2012, 2014, 2016, 2018, and 2020 were selected for this study. By 2020, the survey sites had expanded from the original 25 provinces and 649 villages to more than 3,000 villages in 31 provinces. New homes and people are included in every survey because of changes in home composition and the presence of new members.

In June 2024, CFPS data for children aged 2 to 18 years between 2010 and 2020 were taken out. After initially including 52,953 records based on age, 4,019 that lacked height, sex, or mass information were removed. Then, 292 records were removed due to extremes in height, weight, or BMI, which are determined by adjusting z-scores for age and sex (<−5 or >+5).^16^ There were 8,724 in 2010, 8,133 in 2012, 8,348 in 2014, 8,338 in 2016, 8,193 in 2018, and 6,906 in 2020 records, for a total of 48,642 records. Sample sizes were generally consistent across sex and age. The Peking University Health Science Center’s Medical Research Ethics Committee (IRB00001052-14010) examined and authorized the studies that involved human subjects. Each participant’s legal representatives or next of kin gave written informed consent to take part in this study.

### Measures and definitions

Investigators were recruited from the public before the commencement of each survey and had to complete training before they could take part. The CFPS has combined field trips and phone calls since 2012. Height and mass were reported for children by their parents/guardians or by themselves. BMI was calculated as body weight (kg)/height^2^ (m^2^). The International Obesity Task Force (IOTF) age-sex-specific BMI thresholds were applied to classify participants into: thinness (equivalent to adult BMI <18.5 kg/m^2^), overweight (≥25 kg/m^2^), or obesity (≥30 kg/m^2^).^17^ A published SPSS code was used for the calculations.^18^ Prevalence estimates of overweight always include obesity.

### Statistical analyses

The prevalence was expressed as the sample size and percentage. To make the prevalence comparable across years, population-weighted procedures were used to estimate the overall prevalence. First, the prevalence was calculated by sex and age and then was weighted by the sex- and age-specific population of 2020. The 2020 Chinese Population Census provided the demographic information.^19^ This census in China, which is carried out every 10 years, is the biggest and most reliable assessment of the population situation. Trends in the weighted prevalence of thinness, overweight, and obesity were estimated using linear regression from 2010 to 2018 and from 2010 to 2020, respectively. The regression models from 2010 to 2018 were used as predictive models to calculate the prevalence and 95% prediction intervals for 2020 and to compare them with the observed values for 2020. The *t*-value was obtained by dividing the difference between the observed values minus the predicted values by the standard deviation of the predictions for 2020. A two-sided *P*-value comparing the predicted values to the observed values was obtained by comparing it to a *t*-distribution with three degrees of freedom. Sex and age disparities in trends were further explored by calculating the prevalence at ages 2–6, 7–9, 10–12, 13–15, and 16–18 years for both sexes, boys and girls, respectively, using the same population-weighted procedures. Trends were estimated following the same method. All the statistical analyses were performed by SPSS 29.0 (IBM, Armonk, NY, USA) and GraphPad Prism 10.2.3 (GraphPad Software, Boston, MA, USA).

## RESULTS

### Thinness from 2010 to 2020 by sex and age

**Figure 1.**
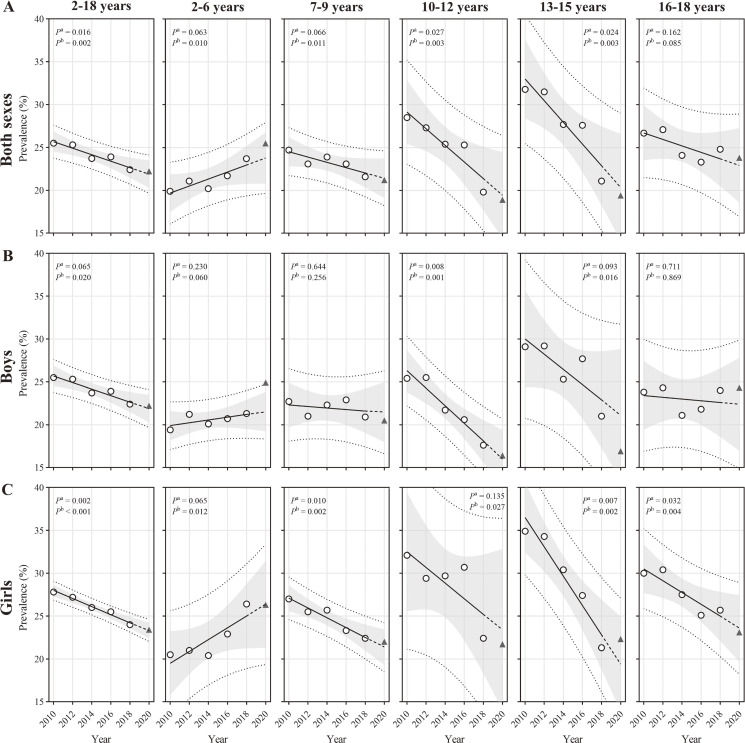
Population-based trends in thinness among Chinese children from 2010 to 2020 according to IOTF BMI cut-offs by sex and age. (**A**) Both sexes; (**B**) boys; and (**C**) girls. Thinness was defined as IOTF equivalent to adult BMI <18.5 kg/m^2^. The sex- and age-specific prevalence were first calculated and then were weighted by the population stratified by sex and age in 2020. The population data were extracted from the Seventh National Population Census of China (2020). Trends were estimated from 2010–2018 and 2010–2020, respectively, using linear regression. *P*^a^ represents the *P*-value for the 2010–2018 linear trend, while *P*^b^ corresponds to the 2010–2018 linear trend. The hollow circles represent prevalence from 2010–2018. The black solid lines represent the fitted line from 2010–2018, and the extended dotted lines represent the projected value for 2020. The triangles represent the observed values for 2020. The grey areas represent the 95% CIs of the fitted line from 2010–2018, and the dotted lines above and below represent the 95% prediction intervals. BMI, body mass index; CI, confidence interval; IOFT, International Obesity Task Force.

eTable 1 shows the crude prevalence of thinness by sex and age from 2010 to 2020. From 2010 to 2020, the weighted prevalence of thinness decreased from 25.5% to 22.2% (*P* < 0.01). Among children aged 7–9, 10–12, and 13–15 years, the prevalence decreased from 24.7%, 28.5%, and 31.8% to 21.2%, 18.9%, and 19.4%, respectively. Nevertheless, among children aged 2–6 years, it increased from 19.9% to 25.5% (*P* < 0.05). No changes were observed among those aged 16–18 years. Figure 1 and eTable 2 show that while sex-specific trends in each age category were consistent with those of the overall population, there was no significant trend among boys aged 2–6 years, while among girls aged 16–18 years, the prevalence decreased from 30.0% to 23.1% (*P* < 0.01). The observed prevalence in 2020 for all subgroups was within the 95% prediction interval of the projected prevalence in 2020 (Table 1).

**Table 1.** Projected and observed prevalence of thinness, overweight, and obesity in Chinese children in 2020 by sex and age

Age categories, years	Thinness (%)					Overweight (%)					Obesity (%)				
		
	Predicted 2020	SD	Observed 2020	*t*	*P*	Predicted 2020	SD	Observed 2020	*t*	*P*	Predicted 2020	SD	Observed 2020	*t*	*P*
Both sexes															
Total	21.9	0.74	22.2	0.405	0.713	23.7	0.85	22.5	−1.412	0.253	11.0	1.17	10.0	−0.855	0.455
2–6 years	23.8	1.38	25.5	1.230	0.305	32.9	2.15	29.4	−1.628	0.202	21.8	2.68	19.0	−1.045	0.373
7–9 years	21.4	1.07	21.2	−0.188	0.864	29.7	2.84	28.4	−0.458	0.678	14.5	1.85	12.6	−1.027	0.380
10–12 years	19.4	2.32	18.9	−0.215	0.844	23.7	3.26	23.5	−0.061	0.955	6.5	1.11	7.1	0.541	0.626
13–15 years	20.3	2.88	19.4	−0.313	0.775	13.6	0.62	15.4	2.903	0.062	2.2	0.38	2.6	1.053	0.370
16–18 years	22.9	2.00	23.8	0.450	0.683	10.3	1.54	9.4	−0.584	0.600	2.2	0.54	1.4	−1.481	0.235
Boys															
Total	20.6	1.07	21.1	0.466	0.673	26.2	1.1	25.9	−0.273	0.803	12.4	1.46	11.7	−0.479	0.665
2–6 years	21.5	1.07	24.9	3.188	0.050	33.9	2.86	30.8	−1.084	0.358	23.8	3.23	20.3	−1.084	0.358
7–9 years	21.5	1.61	20.5	−0.620	0.579	30.3	3.29	33.1	0.851	0.457	15.2	1.85	15.4	0.108	0.921
10–12 years	16.0	1.55	16.4	0.258	0.813	29.8	3.80	27.9	−0.500	0.651	8.2	2.30	9.2	0.435	0.693
13–15 years	21.2	3.53	16.9	−1.217	0.311	17.5	0.72	20.1	3.611	**0.036**	2.9	1.37	4.3	1.022	0.382
16–18 years	22.4	2.50	24.3	0.760	0.503	12.9	1.69	12.8	−0.059	0.957	3.0	0.63	2.2	−1.270	0.294
Girls															
Total	23.3	0.43	23.4	0.234	0.830	20.6	0.93	18.5	−2.258	0.109	9.4	1.14	8.0	−1.223	0.307
2–6 years	26.3	2.33	26.3	0.000	1.000	31.8	1.64	27.8	−2.434	0.093	19.3	2.47	17.5	−0.729	0.519
7–9 years	21.4	0.95	22.0	0.629	0.574	29.0	4.41	23.1	−1.338	0.273	13.7	2.23	9.3	−1.973	0.143
10–12 years	23.4	4.33	21.7	−0.392	0.721	16.8	2.79	18.6	0.645	0.565	4.5	1.55	4.7	0.129	0.906
13–15 years	19.4	2.55	22.3	1.137	0.338	9.2	2.03	9.9	0.345	0.753	1.4	0.77	0.5	−1.169	0.327
16–18 years	23.6	1.79	23.1	−0.280	0.798	7.3	1.58	5.6	−1.076	0.361	1.2	0.56	0.5	−1.250	0.300

SD, standard deviation.

Population-weighted procedures were used to estimate the prevalence. Age-sex-stratified populations were extracted from the Seventh National Population Census of China (2020). Projected 2020 values were calculated by fitting linear regression models to biennial survey data in 2010–2018. Projections for 2020 were estimated using linear regression on data from 2010 to 2018. The *t*-value was obtained by dividing the difference between the observed minus predicted values by the SD of the predictions for 2020. A two-sided *P*-value comparing the predicted values to the observed values was obtained by comparing it to a *t*-distribution with three degrees of freedom. Bold represents statistical significance.

### Overweight from 2010 to 2020 by sex and age

**Figure 2.**
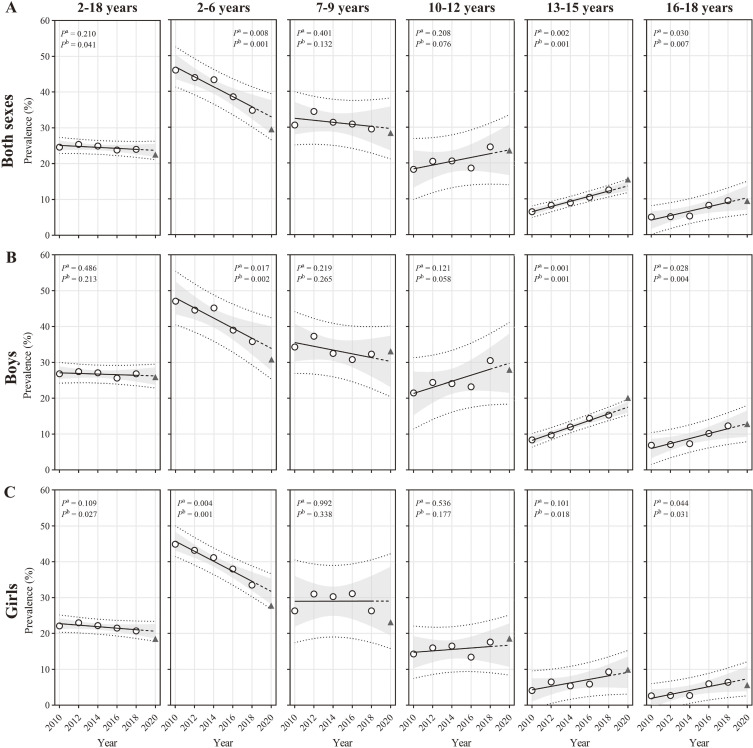
Population-based trends in overweight among Chinese children from 2010 to 2020 according to IOTF BMI cut-offs by sex and age. (**A**) Both sexes; (**B**) boys; and (**C**) girls. Overweight was defined as IOTF equivalent to adult BMI ≥25 kg/m^2^. Overweight includes obesity. The sex- and age-specific prevalence were first calculated and then were weighted by the population stratified by sex and age in 2020. The population data were extracted from the Seventh National Population Census of China (2020). Trends were estimated from 2010–2018 and 2010–2020, respectively, using linear regression. *P*^a^ represents the *P*-value for the 2010–2018 linear trend, while *P*^b^ corresponds to the 2010–2018 linear trend. The hollow circles represent prevalence from 2010–2018. The black solid lines represent the fitted line from 2010–2018, and the extended dotted lines represent the projected value for 2020. The triangles represent the observed values for 2020. The grey areas represent the 95% CIs of the fitted line from 2010–2018, and the dotted lines above and below represent the 95% prediction intervals. BMI, body mass index; CI, confidence interval; IOFT, International Obesity Task Force.

eTable 1 shows the crude prevalence of overweight by sex and age from 2010 to 2020. The weighted prevalence of overweight in the total population decreased from 24.6% to 22.5% (*P* < 0.05). Among children aged 2–6 years, the prevalence decreased from 46.0% to 29.4%; however, among children aged 13–15 and 16–18 years, it increased from 6.4% and 4.9% to 15.4% and 9.4%, respectively (*P* < 0.05), whereas among those aged 7–12 years, it did not change significantly. Subgroup trends by sex within each age category demonstrated concordance with total population estimates, but there was no significant trend among overall boys (Figure 2 and eTable 2). Only the observed prevalence (20.1%) among boys aged 13–15 years was outside the 95% prediction interval for the predicted prevalence in 2020 (17.5%) (*P* < 0.05) (Table 1).

### Obesity from 2010 to 2020 by sex and age

**Figure 3.**
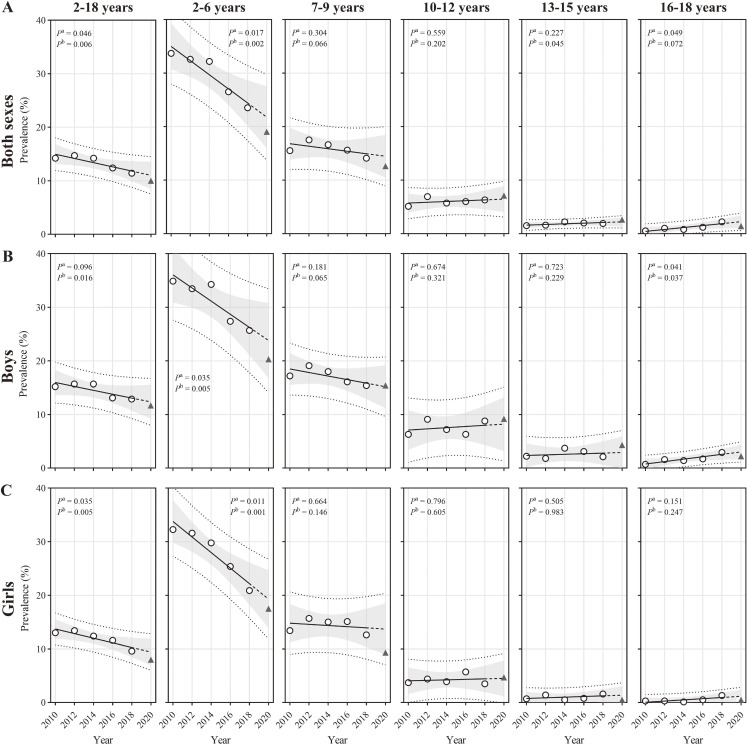
Population-based trends in obesity among Chinese children from 2010 to 2020 according to IOTF BMI cut-offs by sex and age. (**A**) both sexes; (**B**) boys; and (**C**) girls. Obesity was defined as IOTF equivalent to adult BMI ≥30 kg/m^2^. The sex- and age-specific prevalence were first calculated and then were weighted by the population stratified by sex and age in 2020. The population data were extracted from the Seventh National Population Census of China (2020). Trends were estimated from 2010–2018 and 2010–2020, respectively, using linear regression. *P*^a^ represents the *P*-value for the 2010–2018 linear trend, while *P*^b^ corresponds to the 2010–2018 linear trend. The hollow circles represent prevalence from 2010 to 2018. The black solid lines represent the fitted line from 2010–2018, and the extended dotted lines represent the projected value for 2020. The triangles represent the observed values for 2020. The grey areas represent the 95% CIs of the fitted line from 2010–2018, and the dotted lines above and below represent the 95% prediction intervals. BMI, body mass index; CI, confidence interval; IOFT, International Obesity Task Force.

eTable 1 shows the crude prevalence of obesity by sex and age from 2010 to 2020. Between 2010 and 2020, the general population’s weighted prevalence of obesity decreased from 14.2% to 10.0% (*P* < 0.01). Among children aged 2–6 years, the prevalence decreased from 33.7% to 19.0%, while among those aged 13–15 years, it increased from 1.5% to 2.6% (*P* < 0.05). Trends of prevalence remained stable among children aged 7–12 and 16–18 years. Sex-stratified trends across most age categories were consistent with those of the total population, but there were no significant trends in prevalence among children aged 13–15 years in both sexes and an increase in prevalence among boys aged 16–18 years (*P* < 0.05) (Figure 3 and eTable 2). The observed prevalence for all subgroups was within the 95% prediction interval of the projected prevalence in 2020 (Table 1).

## DISCUSSION

This paper revealed a decrease in the prevalence of thinness, overweight, and obesity among Chinese children aged 2–18 years between 2010 and 2020. However, international surveillance revealed divergent patterns. Brazil in 2007–2019 and Poland in 2010–2020 both maintained a stable prevalence of overweight.^20^^,^^21^ Recently, the detection rates of overweight and obesity have risen in Norway,^16^ in the United States,^22^^,^^23^ in South Korea,^24^ in Japan,^25^ and in South Africa^26^ and decreased in Switzerland^27^ and in Spain.^28^ A systematic review including 39 European countries reported that the prevalence of overweight in the 21st century’s first decade was 17.1% and 17.3% for girls and boys, respectively, whereas the prevalence in the second decade was 17.9% and 18.3%, respectively, with a slight increase; the same trend was found in the prevalence of obesity.^29^

Other larger-scale surveys in China have reported results that are not entirely consistent with those of this study. The Chinese National Surveillance on Students’ Constitution and Health data demonstrate that detection rates of overweight and obesity increased over 30 years while that of thinness declined.^7^^,^^8^ Notably, this trajectory reversed in 2014–2019 among children aged 7–9 years, with observed decreases in detection rates of overweight and obesity.^8^ Between 1991 and 2015, the China Health and Nutrition Survey also reported the same upward trend in overnutrition.^30^ This study innovatively encompassed the full pediatric spectrum from ages 2–18 years and added the most recent data from 2020 to estimate population-standardized trends. The difference in age range may be an important reason why the trends of this finding differ from those of other reports, as the steepest declines occurred among preschoolers aged 2–6 years, contributing disproportionately to an overall decline in the prevalence of overweight and obesity. The method of estimation and differences in the definitions of the BMI cut-offs may also contribute to the differences in trends.

Almost all studies reported a declining prevalence of thinness in China. China’s rapid economic growth and accelerated urbanization were closely associated with this nutritional transition.^7^^,^^8^ Throughout the last few decades, Chinese children’s diet patterns have changed from plant-based to animal- and plant-based, with an increase in fat energy consumption.^31^ Concurrent with dietary transitions, China’s rural impoverished population plummeted from 250 million to 70.17 million between 1978 and 2014. Moreover, the population’s proportion of living in poverty dropped from 30.7% to 7.2%.^32^ The Chinese government has embraced urbanization as a powerful approach to economic development. China’s urbanization rate has been steadily rising over the previous few years. It rose from 50.0% in 2000 to 63.9% in 2020.^33^

Trends in overnutrition demonstrated a large age disparity, with decreases among Chinese children aged 2–6 years (preschool children), stable among those aged 7–12 years, and slight increases among those aged 13–18 years in prevalence. In the United States, children aged 12–19 years have been primarily blamed for the increase in the prevalence of obesity among those aged 2–19 over the last 20 years.^23^ From 2005 to 2017, the percentage of Spanish children aged 2–17 years with obesity declined, with the largest decline occurring in those aged 2–5 years.^28^ When compared to the sharp rise in the detection rates of overnutrition in Chinese children in recent decades,^34^ this paper revealed that the trend has been limited in the last 10 years, which is similar to the findings of other studies.^5^^,^^8^^–^^10^ This cannot be separated from the substantial efforts of the Chinese government. In the past 10 years, some regional departments have relied on physical examinations of children aged 0–6 years, strengthened health education such as complementary food addition and a reasonable diet, and carried out focused monitoring and personalized guidance for children with obesity. Between 2016 and 2020, Beijing, China, made notable progress, reducing the prevalence of obesity by over 10%.^35^ Preschoolers should engage in moderate-to-intense exercises for at least an hour each day and screen time for no more than 1 hour, according to China’s first Exercise Guidelines for Preschool Children (aged 3–6 years).^36^ Promoting students’ health and physical fitness was issued by the Chinese government as a national development strategy for the first time in 2007.^37^ Many actions have been taken since then to enhance students’ health, including reorganizing physical education classes and ensuring an hour of physical activity each day in schools.^8^^,^^37^ After 2010, several studies have reported dropped sedentary time and improved physical activity (PA) levels.^38^^,^^39^ However, the detection rates of overnutrition among those aged 13–18 years still rose slightly. This may be because this population is increasingly receiving academic pressure from junior high school and college entrance exams, being more sedentary and not engaging in PA.^37^

This study found that, only for boys at the ages of 13–15 years, the prevalence of overweight in 2020 exceeded the forecast. Long et al^14^ revealed that, between 2018 and 2021, among Chinese children aged 3–7 years, the greatest detection rates of overnutrition occurred in 2020. Body mass may have increased during the initial COVID-19 lockdown, similar to other Chinese findings for children aged 3–19 years.^13^ The prevalence of overweight and obesity did not significantly change in children aged 2–18 years between 2019 and 2020, according to the Korea National Health and Nutrition Examination Survey results.^24^ Although the prevalence of obesity declined between 2020 and 2021, it in Japan was significantly lower in 2019 than in 2020.^25^ However, data were collected over a short time span and did not consider the effects of trends. Nebiker et al^27^ suggested that the prevalence of overweight was greater than expected in 2021 in Switzerland, from their 2014 to 2020 data to forecast values for the year. Employing analogous methodologies, Norwegian researchers reported significantly elevated risks of overweight or obesity exclusively among younger children during pandemic restrictions, contrasting with stable prevalence in older.^16^ To stop COVID-19 spread, all schools nationwide in 2020 delayed school opening and adopted offline or online classes depending on the severity of the pandemic. This has directly led to changes in children’s lifestyles, such as increased in-home time and screen time, decreased PA, and changed dietary patterns, which are related to obesity.^14^^,^^40^^–^^43^ During the initial pandemic outbreak in China (2019 to 2020), children aged 11–16 years spent more time on their mobile phones (from 0.25–1.50 h/day to 0.33–2.00 h/day), and the percentage of those who participated in moderate to vigorous PA ≥60 min/day declined to 11.7% from 14.4%^40^; the PA time of children decreased by 7.1 min/day, sleep time decreased by 15.1 min/day, and recreational screen time increased by 14.8 min/day.^41^ Liu et al^42^ found significant risks of obesity exclusively for recreational screen exposure during containment measures, with no effects for educational screen exposure, despite both types of screen time increasing. Parents who have children with overweight, normal weight, and obesity experienced higher levels of parental anxiety between 2020 and 2021, and 50.70% of families increased their sugary drink as well as dessert consumption. Confinement measures disrupted household food systems, elevating availability and preference for energy-dense foods in children by creating an unstable family environment, increasing parental pressure, and impairing family functions.^43^ Most Chinese boys^44^ enter puberty between the ages of 13–15 years, which has been associated with obesity.^8^ The obesity-causing environment resulting from lockdown may have had a greater impact on children in this age group. On the other hand, boys, who generally have higher levels of PA than girls do, are more affected by the lockdown. One study noted that after the first lockdown was released in April 2020, the levels of PA among Chinese children improved compared with those during the lockdown.^45^ After the short-term lockdown, people were eager to be physically active, online physical education classes became popular in areas with partial lockdown, and schools proposed parental supervision. The lockdown’s impact was lessened because the CFPS survey starts in the year’s second half, which may have caused people to lose mass if they had returned to regular exercise before the data were collected. In addition, a review has shown that prevention programs are most effective at targeting younger children, especially those younger than 12 years of age.^46^

The study implemented a large-scale 2-year wave cross-sectional framework in China between 2010 and 2020, integrating six nationally representative samples through standardized population proportion sampling. This study not only updated the trend of weight status among Chinese children, but also was the first report using long-term trends in weight status to investigate the impact of COVID-19. By incorporating potential demographic information, weighted regression, as well as post-stratification population-weighed estimates, help to control for sampling bias, and stratified analyses account for the confounding effects of age. There are multiple limitations to this study. First, participant’s height and mass were obtained from individual reports, which may have errors compared with the measured values. Large sample sizes as well as a strong correlation between measured and self-reported values, however, significantly reduced estimation errors. Second, data on variables like PA levels, diet, and socioeconomic status that could affect the weight was not included. This made it impossible to provide thorough explanations of the potential reasons behind the trends. Finally, the CFPS is conducted every 2 years, and the two most recent years for which data are available are 2018 and 2020. A thorough evaluation of the effect of COVID-19 on weight status trends is not possible due to the absence of data for 2019 or after 2020.

### Conclusion

The prevalence of thinness, overweight, and obesity decreased among children aged 2–18 years in China from 2010 to 2020, with large age differences. The prevalence of overnutrition among children aged 2–6 years was the primary cause of the decrease, while prevalence among those aged 7–12 years stabilized and slightly increased among those aged 13–18 years. Furthermore, the lockdown may be associated with weight gain among boys aged 13–15 years. Future policies and interventions need to prioritize older children, and the country should monitor changes in long-term weight status as well as PA among children after the pandemic.

## SUPPLEMENTARY MATERIAL

eTable 1. Crude prevalence of thinness, overweight and obesity among Chinese children from 2010 to 2020

eTable 2. Population-based prevalence of thinness, overweight and obesity among Chinese children from 2010 to 2020
