# Supplementary material for: Trends in Thinness, Overweight, and Obesity Among Chinese Children Aged 2–18 Years Before and During the COVID-19 Pandemic in 2010–2020
Source: J Epidemiol. 2025 Dec 5;35(12):510–7. doi: 10.2188/jea.JE20250061 (PMC12620551; doi:10.2188/jea.JE20250061)
Supplement: Supplementary file 1 [file je-35-510-s001.pdf]

eTable 1. Crude prevalence of thinness, overweight and obesity among Chinese children from 2010 to 2020

Age (yrs) categories	Both sexes					Boys					Girls				
		Thinness	Normal weight	Overweight	Obesity		Thinness	Normal weight	Overweight	Obesity		Thinness	Normal weight	Overweight	Obesity
	N	n (%)	n (%)	n (%)	n (%)	*N*	n (%)	n (%)	n (%)	n (%)	*N*	n (%)	n (%)	n (%)	n (%)
Total															
2010	8,724	2,241 (25.7)	4,359 (50.0)	2,124 (24.3)	1,228 (14.1)	4,573	1,072 (23.4)	2,261 (49.4)	1,240 (27.1)	710 (15.5)	4,151	1,169 (28.2)	2,098 (50.5)	884 (21.3)	518 (12.5)
2012	8,133	2,054 (25.3)	3,966 (48.8)	2,113 (26.0)	1,251 (15.4)	4,238	1,001 (23.6)	2,039 (48.1)	1,198 (28.3)	703 (16.6)	3,895	1,053 (27.0)	1,927 (49.5)	915 (23.5)	548 (14.1)
2014	8,348	1,976 (23.7)	4,190 (50.2)	2,182 (26.1)	1,285 (15.4)	4,378	956 (21.8)	2,165 (49.5)	1,257 (28.7)	752 (17.2)	3,970	1,020 (25.7)	2,025 (51.0)	925 (23.3)	533 (13.4)
2016	8,338	1,965 (23.6)	4,220 (50.6)	2,153 (25.8)	1,193 (14.3)	4,426	980 (22.1)	2,223 (50.2)	1,223 (27.6)	668 (15.1)	3,912	985 (25.2)	1,997 (51.0)	930 (23.8)	525 (13.4)
2018	8,193	1,833 (22.4)	4,313 (52.6)	2,047 (25.0)	1,004 (12.3)	4,365	911 (20.9)	2,241 (51.3)	1,213 (27.8)	604 (13.8)	3,828	922 (24.1)	2,072 (54.1)	834 (21.8)	400 (10.4)
2020	6,906	1,531 (22.2)	3,763 (54.5)	1612 (23.3)	732 (10.6)	3,688	777 (21.1)	1,922 (52.1)	989 (26.8)	458 (12.4)	3,218	754 (23.4)	1,841 (57.2)	623 (19.4)	274 (8.5)
2–6 years															
2010	2,664	529 (19.9)	906 (34.0)	1,229 (46.1)	897 (33.7)	1,439	278 (19.3)	482 (33.5)	679 (47.2)	503 (35.0)	1,225	251 (20.5)	424 (34.6)	550 (44.9)	394 (32.2)
2012	2,641	553 (20.9)	917 (34.7)	1,171 (44.3)	871 (33.0)	1,380	290 (21.0)	466 (33.8)	624 (45.2)	469 (34.0)	1,261	263 (20.9)	451 (35.8)	547 (43.4)	402 (31.9)
2014	2,811	570 (20.3)	1,019 (36.3)	1,222 (43.5)	911 (32.4)	1,500	303 (20.2)	515 (34.3)	682 (45.5)	519 (34.6)	1,311	267 (20.4)	504 (38.4)	540 (41.2)	392 (29.9)
2016	3,073	667 (21.7)	1,214 (39.5)	1,192 (38.8)	823 (26.8)	1,616	331 (20.5)	647 (40.0)	638 (39.5)	451 (27.9)	1,457	336 (23.1)	567 (38.9)	554 (38.0)	372 (25.5)
2018	2,730	652 (23.9)	1,138 (41.7)	940 (34.4)	633 (23.2)	1,462	314 (21.5)	632 (43.2)	516 (35.3)	369 (25.2)	1,268	338 (26.7)	506 (39.9)	424 (33.4)	264 (20.8)
2020	2,255	575 (25.5)	1,018 (45.1)	662 (29.4)	427 (18.9)	1,205	301 (25.0)	535 (44.4)	369 (30.6)	243 (20.2)	1,050	274 (26.1)	483 (46.0)	293 (27.9)	184 (17.5)
7–9 years															
2010	1,445	358 (24.8)	645 (44.6)	442 (30.6)	222 (15.4)	780	177 (22.7)	335 (42.99)	268 (34.4)	134 (17.2)	665	181 (27.2)	310 (46.6)	174 (26.2)	88 (13.2)
2012	1,457	336 (23.1)	620 (42.6)	501 (34.4)	256 (17.6)	775	163 (21.0)	323 (41.7)	289 (37.3)	148 (19.1)	682	173 (25.4)	297 (43.5)	212 (31.1)	108 (15.8)
2014	1,525	365 (23.9)	681 (44.7)	479 (31.4)	252 (16.5)	793	177 (22.3)	359 (45.3)	257 (32.4)	142 (17.9)	732	188 (25.7)	322 (44.0)	222 (30.3)	110 (15.0)
2016	1,607	370 (23.0)	740 (46.0)	497 (30.9)	254 (15.8)	874	200 (22.9)	405 (46.3)	269 (30.8)	143 (16.4)	733	170 (23.2)	335 (45.7)	228 (31.1)	111 (15.1)
2018	1,635	352 (21.5)	800 (48.9)	483 (29.5)	230 (14.1)	869	181 (20.8)	406 (46.7)	282 (32.5)	134 (15.4)	766	171 (22.3)	394 (51.4)	201 (26.2)	96 (12.5)
2020	1,378	292 (21.2)	694 (50.4)	392 (28.4)	173 (12.6)	747	153 (20.5)	347 (46.5)	247 (33.1)	115 (15.4)	631	139 (22.0)	347 (55.0)	145 (23.0)	58 (9.2)
10–12 years															
2010	1,576	451 (28.6)	841 (53.4)	284 (18.0)	79 (5.0)	823	209 (25.4)	438 (53.2)	176 (21.4)	51 (6.2)	753	242 (32.1)	403 (53.5)	108 (14.3)	28 (3.7)
2012	1,287	354 (27.5)	671 (52.1)	262 (20.4)	89 (6.9)	668	169 (25.3)	334 (50.0)	165 (24.7)	62 (9.3)	619	185 (29.9)	337 (54.4)	97 (15.7)	27 (4.4)
2014	1,429	362 (25.3)	765 (53.5)	302 (21.1)	84 (5.9)	775	169 (21.8)	416 (53.7)	190 (24.5)	57 (7.4)	654	193 (29.5)	349 (53.4)	112 (17.1)	27 (4.1)
2016	1,355	342 (25.2)	763 (56.3)	250 (18.5)	79 (5.8)	732	151 (20.6)	414 (56.6)	167 (22.8)	44 (6.0)	623	191 (30.7)	349 (56.0)	83 (13.3)	35 (5.6)
2018	1,444	286 (19.8)	803 (55.6)	355 (24.6)	93 (6.4)	768	135 (17.6)	398 (51.8)	235 (30.6)	69 (9.0)	676	151 (22.3)	405 (59.9)	120 (17.8)	24 (3.6)

2020	1,271	238 (18.7)	733 (57.7)	300 (23.6)	91 (7.2)	692	113 (16.3)	386 (55.8)	193 (27.9)	64 (9.2)	579	125 (21.6)	347 (59.9)	107 (18.5)	27 (4.7)
13–15 years															
2010	1,664	534 (32.1)	1027 (61.7)	103 (6.2)	23 (1.4)	824	240 (29.1)	516 (62.6)	68 (8.3)	17 (2.1)	840	294 (35.0)	511 (60.8)	35 (4.2)	6 (0.7)
2012	1,392	439 (31.5)	839 (60.3)	114 (8.2)	22 (1.6)	731	212 (29.0)	447 (61.1)	72 (9.8)	13 (1.8)	661	227 (34.3)	392 (59.3)	42 (6.4)	9 (1.4)
2014	1,391	385 (27.7)	886 (63.7)	120 (8.6)	29 (2.1)	716	181 (25.3)	450 (62.8)	85 (11.9)	26 (3.6)	675	204 (30.2)	436 (64.6)	35 (5.2)	3 (0.4)
2016	1,148	315 (27.4)	712 (62.0)	121 (10.5)	24 (2.1)	618	170 (27.5)	358 (57.9)	90 (14.6)	20 (3.2)	530	145 (27.4)	354 (66.8)	31 (5.8)	4 (0.8)
2018	1,346	284 (21.1)	891 (66.2)	171 (12.7)	26 (1.9)	723	151 (20.9)	459 (63.5)	113 (15.6)	16 (2.2)	623	133 (21.3)	432 (69.3)	58 (9.3)	10 (1.6)
2020	1,156	225 (19.5)	753 (65.1)	178 (15.4)	29 (2.5)	609	104 (17.1)	382 (62.7)	123 (20.2)	26 (4.3)	547	121 (22.1)	371 (67.8)	55 (10.1)	3 (0.5)
16–18 years															
2010	1,375	369 (26.8)	940 (68.4)	66 (4.8)	7 (0.5)	707	168 (23.8)	490 (69.3)	49 (6.9)	5 (0.7)	668	201 (30.1)	450 (67.4)	17 (2.5)	2 (0.3)
2012	1,356	372 (27.4)	919 (67.8)	65 (4.8)	13 (1.0)	684	167 (24.4)	469 (68.6)	48 (7.0)	11 (1.6)	672	205 (30.5)	450 (67.0)	17 (2.5)	2 (0.3)
2014	1,192	294 (24.7)	839 (70.4)	59 (4.9)	9 (0.8)	594	126 (21.2)	425 (71.5)	43 (7.2)	8 (1.3)	598	168 (28.1)	414 (69.2)	16 (2.7)	1 (0.2)
2016	1,155	271 (23.5)	791 (68.5)	93 (8.1)	13 (1.1)	586	128 (21.8)	399 (68.1)	59 (10.1)	10 (1.7)	569	143 (25.1)	392 (68.9)	34 (6.0)	3 (0.5)
2018	1,038	259 (25.0)	681 (65.6)	98 (9.4)	22 (2.1)	543	130 (23.9)	346 (63.7)	67 (12.3)	16 (2.9)	495	129 (26.1)	335 (67.7)	31 (6.3)	6 (1.2)
2020	846	201 (23.8)	565 (66.8)	80 (9.5)	12 (1.4)	435	106 (24.4)	272 (62.5)	57 (13.1)	10 (2.3)	411	95 (23.1)	293 (71.3)	23 (5.6)	2 (0.5)

Note: Thinness, overweight and obesity were defined as International Obesity Task Force (IOTF) <18.5, IOTF ≥25, and IOTF ≥30 kg/m^2, respectively. Overweight includes obesity

eTable 2. Population-based prevalence of thinness, overweight and obesity among Chinese children from 2010 to 2020

Age categories, years	Both sexes					Boys					Girls				
		Thinness	Normal weight	Overweight	Obesity		Thinness	Normal weight	Overweight	Obesity		Thinness	Normal weight	Overweight	Obesity
	N	%	%	%	%	*N*	%	%	%	%	*N*	%	%	%	%
Total															
2010	8,724	25.5	49.9	24.6	14.2	4,573	23.5	49.7	26.8	15.2	4,151	27.8	50.1	22.1	13.0
2012	8,133	25.3	49.2	25.4	14.7	4,238	23.8	48.8	27.4	15.7	3,895	27.2	49.8	23.0	13.4
2014	8,348	23.7	51.4	24.9	14.2	4,378	21.8	51.1	27.1	15.7	3,970	26.0	51.8	22.2	12.4
2016	8,338	23.9	52.4	23.8	12.4	4,426	22.5	51.9	25.6	13.1	3,912	25.5	53.0	21.5	11.6
2018	8,193	22.4	53.6	24.0	11.4	4,365	21.0	52.2	26.8	12.9	3,828	24.0	55.4	20.7	9.6
2020	6,906	22.2	55.3	22.5	10.0	3,688	21.1	53.0	25.9	11.7	3,218	23.4	58.0	18.5	8.0
B^a		-0.38*	0.53*	-0.14	-0.40*		-0.32	0.41*	-0.09	-0.36		-0.47**	0.69*	-0.22	-0.43*
B^b		-0.36**	0.59**	-0.23*	-0.47**		-0.28*	0.39**	-0.11	-0.41*		-0.46***	0.82**	-0.37*	-0.53
2–6 years															
2010	2,664	19.9	34.0	46.0	33.7	1,439	19.4	33.6	47.1	34.9	1,225	20.5	34.6	44.9	32.3
2012	2,641	21.1	35.0	43.9	32.6	1,380	21.2	34.2	44.6	33.5	1,261	21.0	35.8	43.2	31.6
2014	2,811	20.2	36.4	43.3	32.2	1,500	20.1	34.7	45.2	34.3	1,311	20.4	38.4	41.2	29.8
2016	3,073	21.7	39.8	38.5	26.5	1,616	20.7	40.3	39.0	27.4	1,457	22.9	39.1	38.0	25.4
2018	2,730	23.7	41.6	34.7	23.5	1,462	21.3	42.9	35.8	25.7	1,268	26.4	40.1	33.5	20.9
2020	2,255	25.5	45.1	29.4	19.0	1,205	24.9	44.3	30.8	20.3	1,050	26.3	45.9	27.8	17.5
B^a		0.41	1.00**	-1.40**	-1.33*		0.16	1.24*	-1.41*	-1.23*		0.69	0.72**	-1.40**	-1.45*
B^b		0.53*	1.12**	-1.65**	-1.52**		0.41	1.22**	-1.63**	-1.48**		0.68*	1.00**	-1.68**	-1.58**
7–9 years															
2010	1,445	24.7	44.7	30.6	15.5	780	22.7	43.0	34.3	17.2	665	27.0	46.7	26.3	13.4
2012	1,457	23.1	42.6	34.4	17.5	775	21.0	41.8	37.3	19.1	682	25.5	43.5	31.0	15.7
2014	1,525	23.9	44.7	31.4	16.6	793	22.3	45.3	32.5	18.0	732	25.7	44.0	30.3	15.0
2016	1,607	23.1	46.0	30.9	15.6	874	22.9	46.3	30.8	16.1	733	23.3	45.6	31.1	15.1
2018	1,635	21.6	48.9	29.5	14.1	869	20.9	46.8	32.3	15.4	766	22.4	51.3	26.3	12.6
2020	1,378	21.2	50.4	28.4	12.6	747	20.5	46.4	33.1	15.4	631	22.0	54.9	23.1	9.3
B^a		-0.31	0.59	-0.29	-0.24		-0.09	0.61*	-0.53	-0.33		-0.57*	0.57	0.01	-0.11
B^b		-0.33*	0.70*	-0.37	-0.37		-0.15	0.47*	-0.32	-0.31		-0.52**	0.94	-0.42	-0.42

10–12 years															
2010	1,576	28.5	53.3	18.2	5.1	823	25.4	53.1	21.5	6.3	753	32.1	53.5	14.3	3.7
2012	1,287	27.3	52.3	20.5	6.9	668	25.5	50.2	24.4	9.1	619	29.4	54.6	16.0	4.4
2014	1,429	25.4	54.0	20.6	5.7	775	21.7	54.2	24.1	7.2	654	29.7	53.8	16.5	3.9
2016	1,355	25.3	56.1	18.6	6.0	732	20.6	56.2	23.2	6.3	623	30.7	55.9	13.4	5.7
2018	1,444	19.8	55.7	24.5	6.3	768	17.6	51.9	30.5	8.8	676	22.4	60.0	17.6	3.5
2020	1,271	18.9	57.6	23.5	7.1	692	16.4	55.7	27.9	9.2	579	21.7	59.8	18.6	4.7
B^a		-0.97*	0.43	0.54	0.08		-1.03**	0.18	0.84	0.11		-0.91	0.72	0.20	0.05
B^b		-1.01**	0.48*	0.52	0.12		-1.00***	0.29	0.71	0.18		-1.03*	0.71*	0.33	0.06
13–5 years															
2010	1,664	31.8	61.8	6.4	1.5	824	29.1	62.5	8.4	2.2	840	34.9	60.9	4.1	0.7
2012	1,392	31.5	60.3	8.2	1.6	731	29.2	61.2	9.7	1.8	661	34.3	59.2	6.5	1.4
2014	1,391	27.7	63.4	8.9	2.2	716	25.3	62.7	12.0	3.7	675	30.4	64.2	5.4	0.5
2016	1,148	27.6	62.0	10.4	2.0	618	27.7	57.9	14.4	3.1	530	27.4	66.7	5.9	0.8
2018	1,346	21.1	66.3	12.5	1.9	723	21.0	63.7	15.3	2.1	623	21.3	69.3	9.3	1.6
2020	1,156	19.4	65.2	15.4	2.6	609	16.9	62.9	20.1	4.3	547	22.3	67.8	9.9	0.5
B^a		-1.27*	0.54	0.72**	0.06		-0.89	-0.05	0.93**	0.01		-1.71**	1.22*	0.49	0.06
B^b		-1.33*	0.48	0.85***	0.09*		-1.19*	0.07	1.11***	0.15		-1.50**	0.96*	0.54*	-0.001
16–18 years															
2010	1,375	26.7	68.4	4.9	0.5	707	23.8	69.3	6.9	0.7	668	30.0	67.4	2.6	0.3
2012	1,356	27.1	67.8	5.0	1.0	684	24.3	68.7	7.1	1.6	672	30.4	66.9	2.7	0.3
2014	1,192	24.1	70.7	5.2	0.8	594	21.1	71.6	7.3	1.4	598	27.5	69.8	2.7	0.1
2016	1,155	23.3	68.4	8.2	1.2	586	21.8	68.0	10.2	1.7	569	25.1	68.9	6.0	0.5
2018	1,038	24.8	65.7	9.5	2.2	543	24.0	63.7	12.3	2.9	495	25.7	67.9	6.4	1.3
2020	846	23.8	66.8	9.4	1.4	435	24.3	62.9	12.8	2.2	411	23.1	71.3	5.6	0.5
B^a		-0.38	-0.24	0.62*	0.18*		-0.11	-0.60	0.70*	0.23*		-0.70*	0.15	0.55*	0.11
B^b		-0.32	-0.24	0.56**	0.12		0.03	-0.72	0.69**	0.17*		-0.73**	0.31	0.42*	0.06

Note: Thinness, overweight and obesity were defined as International Obesity Task Force (IOTF) <18.5, IOTF ≥25, and IOTF ≥30 kg/m^2, respectively. Overweight includes obesity. Population-weighted procedures were used to estimate the prevalence. The sex- and

age-specific prevalence were first calculated and then weighted by the sex- and age-specific population in 2020. The population data are obtained from the 2020 Population Census in China. Trends were estimated from 2010 to 2018 and 2010 to 2020, respectively, using linear regression. B^a represents the *B* value of the slope from 2010 to 2018, and B^b represents the *B* value of the slope from 2010 to 2020. * represents $P<0.05$, ** represents $P<0.01$, and *** represents $P<0.001$.
